# Supplementary material for: Effects of Liupao Tea with Different Years of Aging on Glycolipid Metabolism, Body Composition, and Gut Microbiota in Adults with Obesity or Overweight: A Randomized, Double-Blind Study
Source: Foods. 2025 Mar 3;14(5):866. doi: 10.3390/foods14050866 (PMC11898661; doi:10.3390/foods14050866)
Supplement: Supplementary file 1 [file foods-14-00866-s001.zip › foods-3480396-supplementary.pdf]

Table S1. The content of main water-soluble components in Liupao tea with different aged years[a].

Component	1 year-aged LPT	4 year-aged LPT	7 year-aged LPT	10 year-aged LPT
Tea Polyphenols, %	12.5	12.0	11.7	11.0
Caffeine, %	6.5	6.4	6.4	6.3
Flavonoids, %	2.4	2.9	3.2	2.2
Catechins, %	2.9	2.7	2.7	2.5
Theabrownin, %	4.7	5.4	6.3	6.3
Thearubigin, %	6.3	6.9	3.7	4.2
Tea Polysaccharide, %	3.2	3.1	1.9	3.3
Free Amino Acids, %	2.4	2.1	1.4	1.3
Soluble Dietary Fiber, %	6.4	5.4	10.2	12.0

[a]All component contents are expressed as percentages (%).

Table S2. The effects of Liupao tea intervention on liver health biomarkers[a]

liver health biomarkers	1-Year Aged Group (n=26)			4-Year Aged Group (n=28)			7-Year Aged Group (n=26)			10-Year Aged Group (n=26)		
	Baseline	Follow-up	*P*	Baseline	Follow-up	*P*	Baseline	Follow-up	*P*	Baseline	Follow-up	*P*
T-BIL, μmol/L	15.85±5.07	14.87±5.28	.136	14.36±5.52	15.26±5.16	.977	14.53±5.86	13.83±3.46	.612	13.83±3.46	14.28±4.58	.676
ALT, U/L	20.17±9.25	18.49±7.91	.147	19.49±8.95	20.90±13.97	.892	23.40±25.08	26.75±32.56	.267	26.75±32.56	19.67±13.14	.838
AST, U/L	23.72±8.19	21.92±6.28	.067	20.75±4.10	22.48±8.08	.551	23.69±13.63	24.00±12.85	.458	24.00±12.85	19.75±6.28	.528
ALP, U/L	66.96±20.28	70.21±21.13	.204	71.5±18.92	71.39±18.85	.178	76.77±24.17	74.32±20.92	.201	74.32±20.92	73.17±18.38	.591
CK, U/L	82.8±39.93	99.29±61.54	.267	113.00±123.77	169.30±342.32	.357	107.73±100.30	116.59±46.01	.003	116.59±46.01	84.83±34.49	.927
LDH, U/L	160.48±31.29	166.17±29.1	.066	166.11±23.81	177.87±33.48	.145	167.73±30.3	175.59±25.16	.022	175.59±25.16	175.96±25.58	.171

[a]T-BIL: total bilirubin; ALT: alanine aminotransferase; AST: aspartate aminotransferase; ALP: serum alkaline phosphatase; CK: creatine kinase; LDH: lactate dehydrogenase.

Table S3. Intention-to-treat analysis of the intervention effects of Liupao tea with different aging years on metabolic parameters[a]

Metabolic parameters[b]	1-Year Aged Group (n=28)			4-Year Aged Group (n=28)			7-Year Aged Group (n=28)			10-Year Aged Group (n=28)		
	Baseline	Follow-up	*P*	Baseline	Follow-up	*P*	Baseline	Follow-up	*P*	Baseline	Follow-up	*P*
SBP, mmHg	129.04±22.27	121.81±21.7	0.003	132.21±16.31	122.11±17.2	0.002	131.69±14.06	117.77±23.66	0.006	132.08±16.22	122±15.46	<0.001
DBP, mmHg	75.54±13.09	73.96±13.20	0.466	79.04±9.01	74.79±13.11	0.065	78.81±10.45	75.19±16.75	0.214	82.19±14.46	72.92±11.71	<0.001
TC, mmol/L	5.05±1.09	4.88±0.86	0.145	5.07±1.14	4.87±1.11	0.060	5.11±0.91	5.39±0.84	0.112	5.21±0.81	5.00±0.86	0.257
TG, mmol/L	1.25±0.80	1.32±0.88	0.389	1.40±1.25	1.20±0.83	0.266	1.63±0.85	1.90±1.25	0.114	1.72±1.82	1.59±1.59	0.268
HDL-C, mmol/L	1.33±0.36	1.35±0.31	0.481	1.29±0.23	1.36±0.21	0.077	1.27±0.28	1.32±0.27	0.169	1.32±0.23	1.37±0.17	0.132
LDL-C, mmol/L	2.88±0.68	2.66±0.57	0.032	2.93±0.83	2.68±0.76	<0.00	3.02±0.64	2.97±0.61	0.687	2.93±0.51	2.65±0.54	0.014
HbA1c, %	5.58±0.99	5.68±0.99	0.035	5.69±0.81	5.63±0.68	0.625	5.83±1.16	5.97±1.12	0.692	5.75±1.28	5.87±1.37	0.055
FBG, mmol/L	5.96±1.83	5.88±2.09	0.351	5.95±1.56	5.50±1.07	0.022	8.12±11.12	6.12±1.69	0.339	6.02±2.42	5.90±1.97	0.325
INS, pmol/L	89.67±77.39	70.68±43.54	0.233	83.04±59.84	64.28±30.38	0.053	74.42±34.83	72.71±44.54	0.786	83.68±49.52	73.66±45.19	0.089
Weight, kg	69.87±13.56	68.51±13.01	0.006	69.87±12.60	66.7±10.57	0.005	67.83±10.36	65.87±10.45	0.043	68.77±14.09	66.72±13.05	0.004
BMI, kg/m^2	25.94±3.18	25.37±3.08	0.010	26.67±3.76	25.22±2.83	0.032	25.45±2.70	24.63±2.61	0.008	25.81±3.56	25.07±3.31	0.005
LBM, kg	45.19±11.76	46.55±9.23	0.361	47.36±8.95	47.79±9.31	0.137	46.55±7.75	47.68±8.76	0.102	47.00±10.32	47.05±11.15	0.950
BFM, kg	22.65±5.79	20.40±4.82	0.012	22.56±6.61	19.86±5.73	<0.00	21.57±4.76	19.36±4.93	0.002	22.13±5.96	25.48±28.16	0.559
BFP, %	31.85±4.99	30.25±4.45	0.001	32.03±6.62	29.12±6.94	<0.00	31.61±5.19	29.48±5.07	<0.001	31.98±5.61	29.63±5.97	<0.001
VFA, cm^2	112.00±69.37	91.42±34.75	0.073	104.21±39.66	78.89±31.72	<0.00	95.54±28.97	80.81±30.01	<0.001	101.27±39.20	86.04±32.30	<0.001

[a]Missing data from dropouts were handled using Multiple Imputation by Chained Equations (MICE), generating reasonable imputed values for the missing follow-up data based on available baseline information and other covariates. [b]Abbreviations: SBP: Systolic blood pressure; DBP: Diastolic blood pressure; TC: Total cholesterol; TG: Triglycerides; HDL-C: High-density lipoprotein cholesterol; LDL-C: Low-density lipoprotein cholesterol; HbA1: Hemoglobin A1c; FBG: Fasting blood glucose; INS: Insulin; BMI: Body Mass Index; LBM: Lean Body Mass; BFM: Body Fat Mass; BFP: Body Fat Percentage; VFA: Visceral Fat Area.

Table S4. Relative abundance and changes of PICRUSt2-predicted KO functions in Liupao tea of different aging years[a].

KO ID	1-Year Aged Group (n=26)			4-Year Aged Group (n=28)			7-Year Aged Group (n=26)			10-Year Aged Group (n=26)		
	Baseline	Follow-up	*P*	Baseline	Follow-up	*P*	Baseline	Follow-up	*P*	Baseline	Follow-up	*P*
K02529	0.00555±0.00113	0.00510±0.00153	0.248	0.00571±0.00088	0.0053±0.00192	0.355	0.00566±0.00114	0.00532±0.00167	0.442	0.00584±0.00119	0.00574±0.00104	0.753
K02003	0.00509±0.00099	0.00479±0.00135	0.378	0.00516±0.00080	0.00471±0.00163	0.236	0.00495±0.00079	0.00468±0.00137	0.442	0.00519±0.00092	0.00498±0.00096	0.448
K01190	0.00485±0.00109	0.0045±0.00149	0.361	0.00500±0.00117	0.00447±0.00178	0.227	0.00507±0.00103	0.00471±0.00169	0.404	0.00479±0.00107	0.00449±0.00118	0.356
K02004	0.00482±0.00102	0.00445±0.00136	0.293	0.00504±0.00106	0.00457±0.00158	0.231	0.00489±0.00101	0.00461±0.00151	0.476	0.00485±0.00101	0.00464±0.00106	0.468
K01990	0.00448±0.00094	0.00421±0.00127	0.401	0.00458±0.00097	0.00416±0.00148	0.253	0.00436±0.00076	0.00415±0.00135	0.543	0.00452±0.00094	0.00424±0.00089	0.290
K06147	0.00440±0.00077	0.00402±0.00100	0.149	0.00441±0.00082	0.00413±0.00115	0.317	0.00436±0.00068	0.00417±0.00102	0.492	0.00439±0.00085	0.00425±0.00072	0.549
K01992	0.00302±0.00053	0.00300±0.00061	0.880	0.00299±0.00057	0.00288±0.00068	0.547	0.00283±0.00049	0.00299±0.00081	0.445	0.00304±0.00072	0.00289±0.00059	0.430
K03088	0.00280±0.00093	0.0026±0.00097	0.460	0.00267±0.00088	0.00245±0.00105	0.435	0.00247±0.00077	0.00243±0.00111	0.890	0.00276±0.00080	0.00274±0.00100	0.921
K05349	0.00272±0.00054	0.00270±0.00053	0.861	0.00291±0.00059	0.00261±0.00061	0.081	0.00266±0.00035	0.00277±0.00069	0.498	0.00274±0.00045	0.00264±0.00053	0.500
K07024	0.00229±0.00067	0.00216±0.00058	0.461	0.00225±0.00053	0.00217±0.00084	0.703	0.00220±0.00065	0.00216±0.00089	0.863	0.00234±0.00046	0.00238±0.00061	0.812

[a]KO functions were ranked by relative abundance at baseline and follow-up. Top 10 KO IDs were selected. Wilcoxon signed-rank test was used to compare changes within the same samples.

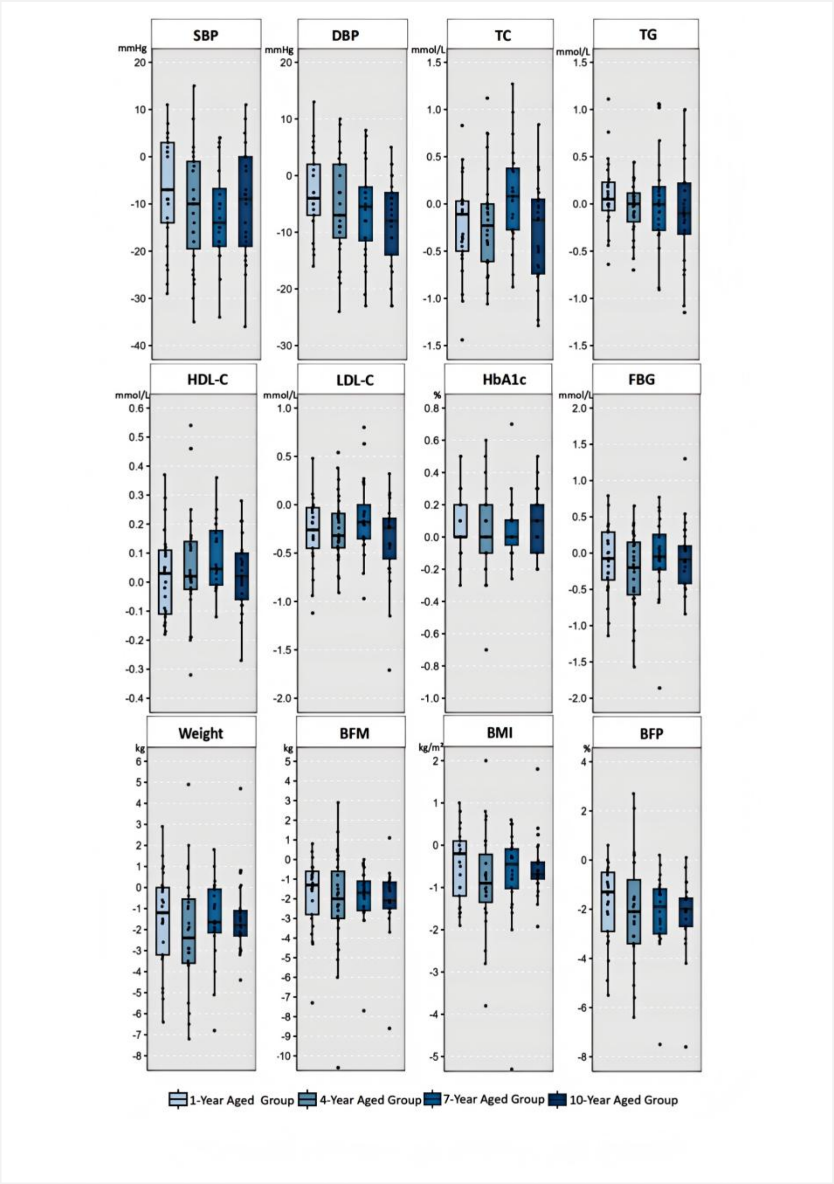

Figure S1. Differences in the effects of Liupao tea with different aging years on improving metabolic parameters and body composition.
